# Real-Time Power Analysis of Smart Sensors Using Advanced Debugging Methods

**DOI:** 10.3390/mi12111276

**Published:** 2021-10-20

**Authors:** Daniel Gis, Nils Büscher, Christian Haubelt

**Affiliations:** Institute of Applied Microelectronics and Computer Engineering, Faculty of Computer Science and Electrical Engineering, University of Rostock, 18059 Rostock, Germany; nils.buescher2@uni-rostock.de (N.B.); christian.haubelt@uni-rostock.de (C.H.)

**Keywords:** power-analysis, power-model, energy-aware, inertial sensors, evaluation, hardware-in-the-loop, repeatability, reproducibility, smart sensor

## Abstract

To achieve a good estimate of the power consumption of an embedded system, including its firmware, is a crucial step in the development of systems with a severely constrained power supply. This is especially true for cases where the device is powered by a small battery or through energy harvesting. The state-of-the-art approaches to measure or estimate the power consumption are formal methods, using power debugging tools with the real hardware or simulation based estimations. In the work at hand, a novel method to estimate the power consumption is proposed, it utilizes the sensor-in-the-loop architecture and enhancing it with a power estimation functionality. The proposed method combines the benefits of former methods, allowing for run-time analysis of the power-consumption in a reproducible way using recorded data without the need for power debugging hardware. In the experiments it is shown that, once set up, the proposed method is able to estimate the power consumption with an error of less than 1% compared to a power debugging hardware. Thus, the proposed method provides a reliable and fast way to estimate the systems power consumption.

## 1. Introduction

When working with embedded systems, very often the power consumption of the used system is limited or should be as low as possible [1,2]. The overall consumption of the system does not only depend on the used hardware but also on the software running on the embedded system. For example, if the system wakes up regularly and conducts complex calculation, the power consumption will be relatively high. Therefore, it is important to have means to easily measure the power consumption of a system. However, when working with embedded systems that incorporate environmental sensors and especially when working with smart sensors, the power profiling can be extremely complicated. For example, when the state of the system can change depending on interrupts coming from the sensors or when the complexity of the calculation depends on the data from the sensors, the power profiling can be very complex and time-consuming. Recently, the Sensor-in-the-Loop (SiL) debugging platform was developed that enables a developer to re-inject previously recorded sensor data back into the sensor to create a repeatable and reproducible way to evaluate the firmware on the smart sensor [3]. In the work at hand, we propose an extension to the SiL platform enabling it to perform a simulated power analysis of the system to estimate its power consumption. With this proposed extension, the SiL platform enables a developer to easily estimate the power consumption of the smart sensors for a given scenario using the previously recorded and re-injected sensor data. Additionally, the added power profiling ability makes it possible to easily optimize the sensor firmware in a fast and targeted way towards lower power consumption. By re-injecting previously recorded sensor data, energy-intensive parts of the firmware can be identified and optimized. Using the pre-recorded data ensures that a changed power consumption is caused by changes in the code and not by differences in the live sensor data. Additionally, the proposed extension to the SiL architecture is also capable to estimate the power consumption while recording live data from the sensors.

The rest of this paper is structured as follows: At first, we will discuss the related work in Section 2. Afterwards, we will describe the concept of our approach in Section 3. This section is separated into two subsections. The first part presents the actual conceptual part of the power aware debugging method and the developed power model. The second part describes the example implementation on a smart inertial sensor. In Section 5, we will show our experimental setup and the different experiments done to investigate the usability of our approach. The experiments will be followed by the results in Section 6. In this section, we will present and discuss the results of our experiments using the example implementation of our power aware debugging approach. The final section, Section 7, concludes this paper and outlines further work in this field.

## 2. Related Work

Currently, there are three state-of-the-art approaches to conduct a power analysis for a system or prototype [2]. All three approaches have their own benefits and limitations. The work at hand presents a novel approach witch combines the benefits of the state-of-the-art methods.

The first state-of-the-art method is to use a power debugger connected to the prototype to measure its power consumption at runtime [4,5]. The great benefit of this approach is that the power is measured for the real hardware and shows all effects including changes in the power consumption from clock drift, temperature changes, or other environmental influences. However, using a power debugger has the drawback, that the measurements are not entirely reproducible when working with live sensor data. Additionally, the power debugger can influence the measured power because it adds an overhead for the required communication (e.g., via JTAG) and might prevent certain power saving states of the Microcontroller (μC) due to the communication.

The second state-of-the-art approach is using a simulation for the power analysis [6,7,8]. This approach allows to do the power analysis for all power states and in a reproducible way. However, for the simulation, the power analysis cannot be done in real-time and highly depends on the accuracy of the model, e.g., cycle accurate [7], instruction accurate [6], or component based [8]. The creation of the simulation model for the power analysis might be time-consuming.

The third state-of-the-art approach is to conduct a formal power analysis of the compiled software for the microcontroller. For example, in [9] the power consumption for each compiler instruction has been determined for the overall power consumption. Said approach also considers the effects of caches, cache misses, or stalls. In [10], a formal analysis for an 8-bit microcontroller has been proposed which also considers the power consumption of peripherals and not only the microcontroller itself. Both methods can reveal particularly power intensive software parts and estimate the rough overall power consumption. However, the analysis is not data dependent making these approaches unsuitable for systems where the state depends on input data. A formal approach including data dependency has been presented in [11] which estimates the worst case power consumption at instruction level. However, often the average power consumption is more relevant than the worst case power consumption. Furthermore, the authors in [11] stated, that a formal analysis of whole complex programs can be very time-consuming. None of the above-mentioned formal methods is able to also analyze the power consumption of connected hardware such as inertial sensors.

Often the manufacturers of microcontroller which are used in embedded systems with low power requirements provide tools or plug-ins for their development Integrated Development Environment (IDE) to help the developers to gain an overview over the power consumption early in the development process [12,13]. These tools normally use one of the above-mentioned methods, or a combination of them for the power estimation.

The approach presented in the work at hand aims to combine the benefits of the state-of-the-art methods by allowing for a power analysis in real-time on the real hardware with a previously created power model of said hardware. Thus, using the SiL architecture it combines the benefit of a live power estimation on the real hardware with the benefit from simulation approaches to allow for reproducible results. The drawback of the proposed approach is that it also requires the creation of a power model.

## 3. Smart Sensor Power-Model

In this section, we will present our approach to extend a state-of-the-art development and debugging environment by an innovative component to generate a power estimation for the whole system. This enables the developer of such system to add energy awareness to his development and testing process.

The power estimation is achieved using two core components providing the necessary functionality. The first component should allow a communication with the examined smart sensor to fulfill two objectives. First it should be possible to observe the internal state and receive data from the smart sensor during runtime or while debugging the sensor. Secondly, the communication component should allow sending data from the host to the sensors, so that said sensor can process previously recorded data. This will lead to a repeatable and even reproducible investigation of the sensor system and the firmware under development. Hence, also to a reproducible way to measure the power consumption for specific situations. With the recently presented sensor-in-the-loop debugging architecture [3], this functionality is already available.

The second core component is a dedicated power model for the smart sensor the firmware is developed for. This power model (PM) fulfills the actual estimation of the power consumption from the smart sensor in use. It must be able to observe the power state of the system. This can be done automatically by observing special power dedicated registers of the system. By observing the power state of the system said power model can report the current power consumption of the system using previously defined power consumption rates in combination with the state. However, in complex smart sensors where not all power related registers are accessible at any time, this can be a challenging task. In this case, it is more practical to use the PM itself to control the power state of the system.

**Figure 1:**
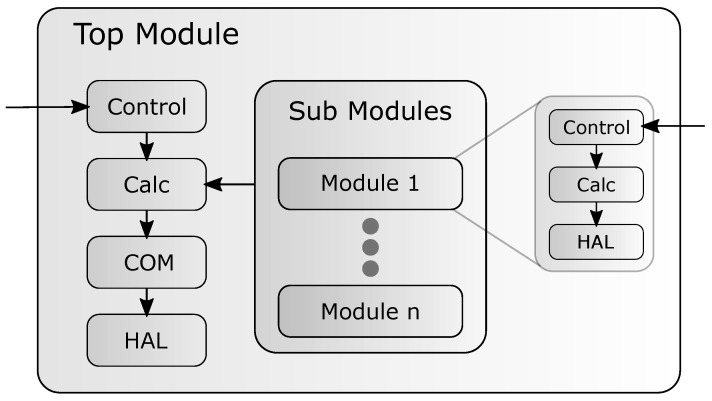
Conceptual structure of the sensor power model.

In this work, we propose such a power model, which is able to control the power states of each system component of the smart sensor and report the power consumption back to the host. Such a power model could be designed as shown in Figure 1. This PM has a hierarchical structure to handle modern complex smart inertial sensor consisting of multiple system components. Usually, a smart sensor has at least one processing unit, which is equipped with a variety of peripherals such as General Purpose Input Outputs (GPIOs), Analog Digital Converters (ADCs), or Comunication Interfaces (CIs). Aside of this so-called Sensor Processing Unit (SPU) there are one or more actual sensors, e.g., accelerometer, gyroscope, magnetometer, or a temperature sensor. Each of these components should be modeled as a separate module in the power model with its own states, power consumptions estimations, and control logic.

The PM of the smart sensor shown in Figure 1 consists of a top-level-module which includes the sub-modules for the different components of the system. Inside the top-module are four functional blocks which handle different tasks. The *control* block handles the communication between the user and the power model. Using this block, the firmware developer should be able to switch power states and find additional information of the model. When the control block triggers a change of the state, the calculation *Calc* block takes over and calculates the estimated power consumption for the new state. Therefore, it requests the current power consumption of each sub-module. This updated power estimate is reported back to the firmware developer by the communication *COM* block of the model. The last functional block configures the selected power mode for the actual hardware component. To configure the actual hardware power state, the hardware abstraction layer block *HAL* is used. It consists of functions or mechanisms to reconfigure the power state of the corresponding hardware element. Each sub-module of the PM functions as an independent component and can be directly configured and accessed from the outside of the PM by the developer. The sub-modules cannot be configured by the top-level module to ensure that there are no unexpected changes in the power state of the components of the system. The functionality of the sub-modules is separated into three functional blocks quite similar to the top-module. The control block manages the state of the sub-module and allows communication. It also communicates with the top-level-module. The functional behavior of the *Calc* block and the *HAL* block is similar to the top-module. When a sub-module changes its power state and calculates the new power estimate, it reports this change to the top module. The top module then recalculates the power consumption of the whole system and reports the changes back to the developer. The *COM* block is missing in the sub-modules since the reporting of the power estimation is handled by the top-level-module.

The implementation of this concept combines the benefits of state-of-the-art methods for power estimation used with smart sensors. It offers a repeatability and reproducibility which is currently only possible using simulation based approaches. In addition to that, this method uses the real hardware to test and verify the sensor firmware. This enables the developer to gain information about functional characteristics and even more important, extra-functional characteristics from the system under test. The extra-functional characteristic power consumption can be observed aside of different others, such as runtime and memory usage. In comparison to methods that measure the power consumption during the debugging process, this method delivers the power numbers that would apply for a running target without a connected debugger.

## 4. Example Implementation

The proposed power model was implemented on the BMF055 [14], a state-of-the art smart inertial sensor. This sensor was used, because it has already an implementation of the sensor-in-the-loop interface [3]. This adds the benefit to easily transfer the estimated power consumption to the firmware IDE. Furthermore, it enables us to use pre-recorded sensor data for reproducible tests of the implemented power model.

We implemented the power model according to the concept described in Section 3 and shown in Figure 1. The C programming language was used to implement the developed PM on the actual sensor firmware. The top-module is represented by the SPU, more specific by the ATSAMD20J18 μC. All blocks are implemented as described in Section 3. For the *COM* block, the SiL interface is used to communicate the estimated power consumption. The *HAL* block contains functions provided by *Microchip* as the manufacturer of the used μC.

**Figure 2:**
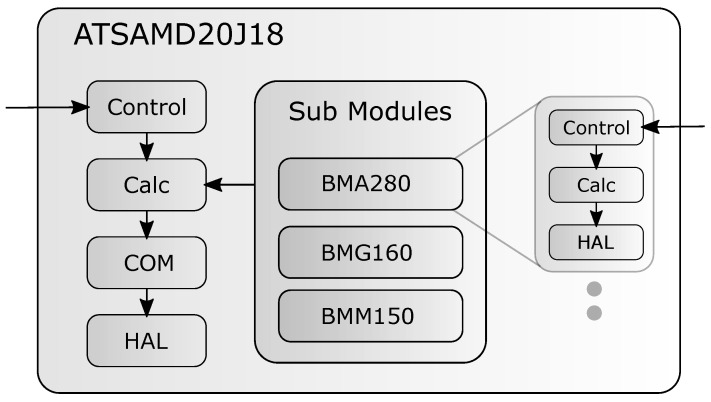
Specific structure of the sensor power model.

The implemented power model has three additional sub-modules, one for each sensor element of the BMF055 smart sensor. The sensor is equipped with an accelerometer (BMA280) [15], a gyroscope (BMG160) [16], and a magnetometer (BMM150) [17]. For each of these sensors a sub-module was created. The *HAL* block of these modules is implemented by using the hardware abstraction library provided by *Bosch Sensortec* [18]. In Figure 2, one can see the specific implementation of the power model on the BMF055 smart sensor.

The initial power values for the individual states of the power model were taken from the data sheets of the individual components of the smart sensor. In Table 1, these numbers are listed for each component separately. The current for the individual components were measured at different voltage level. The data sheet of the sensors delivers measurements at 2.4 V and the microcontroller data sheet at 3.3 V. The sensors have an internal linear voltage regulator, so that the current is independent of the voltage as long as the voltage is in the allowed range. The current of the microcontroller depends on the used voltage, so that we will use the 3.3 V for the whole system in the experiments. That will ensure comparable results. All parts are listed with their individual power states and the corresponding power consumption. As will be discussed in Section 6, these estimates are not very reliable for all use-cases and have to be calibrated to achieve satisfactory results.

**Figure 3:**
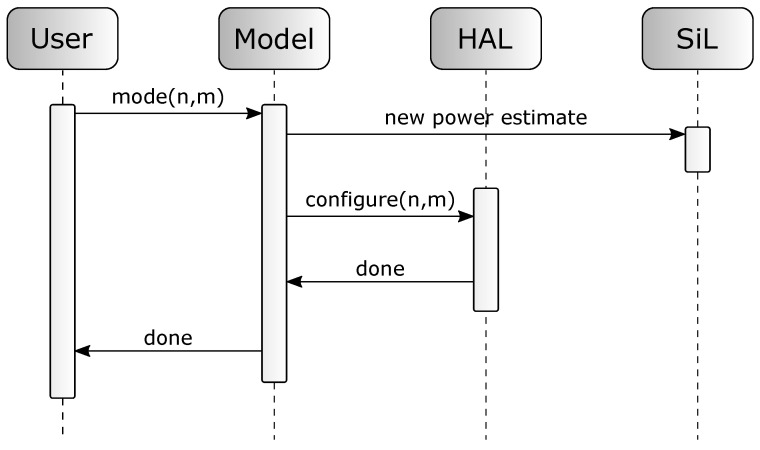
Sequence diagram of user–model interaction.

Figure 3 shows the sequence diagram of a power mode switch. The user configures a new power mode using the *control* block. The module calculates the new power estimate and communicates it using the SiL interface. After that, the *HAL* is invoked by the model to switch the actual power state of the sensor component.

**Figure 4:**
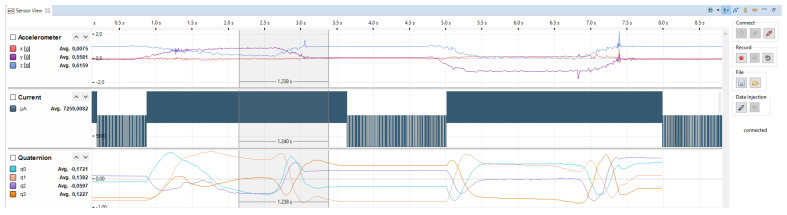
Sensor view in Eclipse environment.

In Figure 4, one can see how the power consumption estimated by the power model will be visualized by the sensor-in-the-loop framework. This figure shows the data for the complex real-world scenario with state changes and different sampling rates of the sensors. A more detailed description of these example can be found in Section 5. In Section 6, more detailed views of the current consumption delivered by the model can be seen. The framework will visualize the current flow into the system, the actual power consumption depends on the voltage level used to power the system. For our experiments we used a voltage level of 3.3 V but that can vary in different scenarios. In addition to the power estimates, the developer can see raw sensor data of each sensor. Furthermore, it is possible to show internal system states or results from sensor algorithms such as the quaternion representation of the attitude of the sensor. Using this, all observable data can set in relationship to the power estimate of the system and enables the developer for an energy aware system development. This screenshot shows a sequence of approximately 8.5 s, to see details of the current signal, the user has to zoom into the signal. A more detailed view of the current signal can be seen in Section 6.

## 5. Experiment

After implementing the power-model on the smart sensor of choice, experiments were conducted for the power consumption of the system. These experiments were separated into two series of measurements:

1. In the first series the power consumption of each individual component of the sensor was measured and compared against its power model. Hence, in this series it can be verified how well the power model fits with the actual hardware. Additionally, these measurements can be used to calibrate the power model for all components to ensure a more accurate power estimation of the whole system. These experiments can also reveal where more attention is required to make the power model as accurate as possible;
2. The second part of the experiments was done for the power estimation of the whole system consisting of the individual components. This experiment should show how well the overall power estimation can be done using the proposed method.

To measure the power consumption of our smart sensor setup, a current waveform analyzer was used [19]. The power supply for the sensor was configured to a fixed voltage level of 3.3 V. This 3.3 V are used to have comparable results to the current values taken from the data sheets. The values for the microcontroller are listed with a voltage of 3.3 V and depends on this voltage. Current values for the sensors are voltage independent regarding their internal voltage regulator.

The measurements were done for different power states, e.g., active, sleep, and standby. During the experiments, the power states of the tested component or system are iterated through, After each power state change of any component of the smart sensor, the internal power model calculates the current power consumption of the sensor. This new power value is sent to the IDE using the Sensor-in-the-Loop interface. In the IDE, these data can be visualized aside of the raw sensor data and the result from the orientation calculation. For our experiments, we compared these data with the actual power consumption measured by the current waveform analyzer. In combination with the information from the data sheets the power models can be calibrated for more accurate results.

### 5.1. Individual Components

For the measurements of the individual components, all enclosed components and sensors of the smart sensor system were configured to their power modes with the least power consumption. This ensures to keep the influence of other components as small as possible. The measurements for all tested components were done using the same methodology. The tested component starts in is default power state. In an interval of two seconds, the tested component switches between all its possible power states. The time of each power state is controlled by a timer which invokes a timer interrupt to wake up the processor. After the wake-up, the next power state is configured and the timer is started again. With a sampling rate of the waveform analyzer of 1 Msamples per second, the two seconds interval provides enough samples to achieve meaningful results for each state.

**Figure 5:**
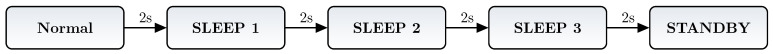
Control flow of SPU modes test.

For the first test of the individual components, the SPU was measured in all possible power states. The SPU used in our smart sensor is a ATSAMD20J18 microcontroller from Microchip [20] The flow chart in Figure 5 shows the program flow of the firmware during the measurements.

After the measurement for the SPU, the power consumption from each inertial sensor was examined. Therefore, the SPU was set into standby mode to minimize its influence.

**Figure 6:**
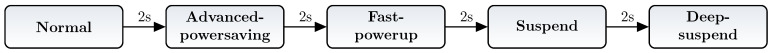
Control flow of gyroscope modes test.

First, the power consumption of the gyroscope was measured. As shown in Figure 6, all possible power modes of this sensor device were configured.

**Figure 7:**
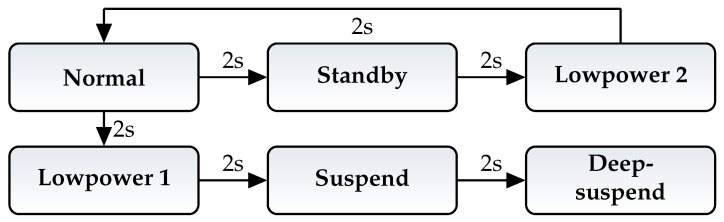
Control flow of accelerometer modes test.

The second measured sensor was the accelerometer. Figure 7 shows the control flow of for the accelerometer measurements

Using the accelerometer it is not possible to switch directly between all power modes. This is not possible because there is no valid state transition between the *lowpower 2* mode and the *lowpower 1* mode. This makes it necessary to switch back to the *normal* mode before using the *lowpower 1* mode. Aside of this, the test is done similar as for the gyroscope.

**Figure 8:**
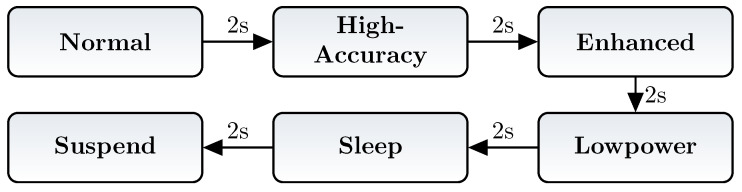
Control flow of magnetometer modes test.

The last measured sensor was the magnetometer. It has the most power modes of all sensor devices used in the smart sensor. The sampling modes are divided into four modes from *normal* to *lowpower*. The measurements were done similar to both previous sensors, the control flow can be found in Figure 8.

After the experiments for the isolated modes of each component of the smart sensor are done, the measured values can be used to compare against the values of the data sheets. Additionally, the results from the measurements are used for the calibration of the power model of the components to achieve more accurate results This step can be found in Section 6.

### 5.2. Measurement of the Whole System

**Figure 9:**
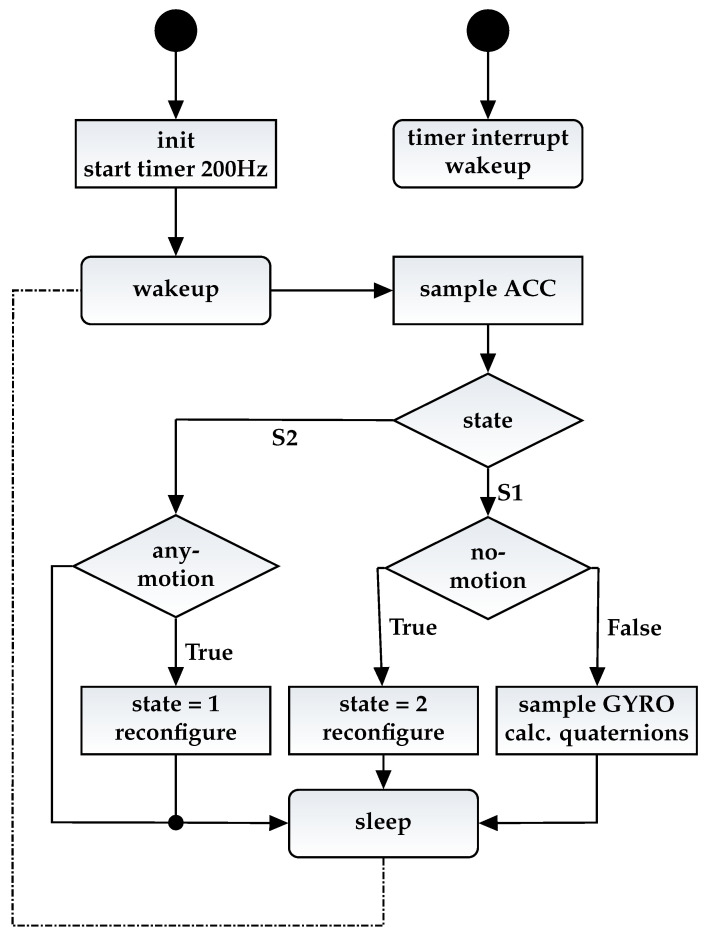
Control flow of complex test case.

After the measurements and calibration for the individual components of the systems, an experiment for the whole system was conducted. This is supposed to verify how well the proposed methodology can model the power consumption using the models for each Individual component. To compare the power consumption of the whole setup against the power values delivered by our power model, we constructed a complex test case. This test case is a commonly used application for smart sensors. The flow chart in Figure 9 describes the program flow of the smart sensor firmware.

The program is mainly partitioned into three phases. The firmware starts with the initialization phase, were the SPU and all peripherals, such as GPIOs, communication interfaces, and timers, are configured. To sample the gyroscopic and the accelerometer data, a timer is configured to fire an interrupt with a frequency of 200 Hz.

The initial state of the firmware is S1, after every interrupt the sensor data are sampled and a “No Motion” algorithm checks if the sensor is moving using the accelerometer data. If the sensor is moving, the orientation of the sensor is calculated using the Madgwick IMU algorithm [21]. This algorithm calculates the orientation of the sensor as a quaternion representation using the angle rates and the acceleration data. The sensor goes into sleep mode, after the determination of the orientation until the next timer interrupt occurs. If the “No Motion” algorithm in S1 detects that the sensor is not moving anymore, the state is switched to S2 and the SPU goes into sleep mode. Furthermore, the gyroscope is configured to the “Fast powerup” sleep mode because its data are not needed in S2. The timer for the sampling rate is reconfigured to 50 Hz.

In S2, an “Any Motion” algorithm detects if the sensor is moving again. For that, the algorithm just uses the 50 Hz accelerometer data. The gyroscope is in a “Fast powerup” sleep mode and no gyroscopic data are sampled in this state. If the “Any Motion” algorithm detects a movement of the sensor, the state is switched back in S1. Furthermore, the gyroscope is switched into “Normal” mode and the update rate of the timer is reconfigured to a 200 Hz sampling frequency.

## 6. Results

In the previous section, we explained the different experiments done on our setup. In this section, we will show the results of these experiments, each result will be discussed in detail.As described in the previews section, the experiments were separated into two series. In the first series, the power consumption for each individual component were measured. These measurements were used to verify the basic practicability and accuracy of the proposed power estimation method. Furthermore, the measurements were used to calibrate the power models of each component.

The second experiments were done after the calibration to measure the accuracy of the proposed power estimation for the whole system.

### 6.1. Individual Components

**Figure 10:**
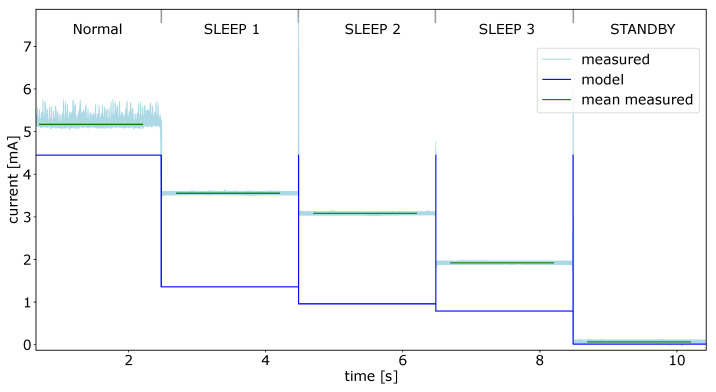
SPU modes power model data and measured current values.

In the first experiment for the Individual components the power consumption for each power mode of the SPU was measured. The results for the measurements of all five power modes are displayed in Figure 10.

The actual measured current is shown in light blue. To find a comparable current value, the mean of the current values over 1.5 s for each power state was calculated. Said mean is drawn in green over the measured current values. The calculated values of the power module are shown in blue, these values represent the mean current for the whole sensor system at a given time. The high peaks between the different states are caused by the enablement of the SPU to reconfigure the power state. During this short period of time the power consumption is much higher than average, because the capacities of the system has to be charged after the enablement [22,23].

One can see, that the output of the power model and the actual measured values do not fit very well considering the current. This has mainly two reasons:

1. The values in the data sheet are measured under very specific conditions regarding the configuration of the actual microcontroller and its peripherals. The power consumption of a SPU or any other microcontroller depends on a variety of factors that determine the setup. It highly depends on the clock setup, for example what component is used as clock (internal clock oscillator or external oscillator). The clock frequency used for the separate clock domains for the core and the peripherals are also a factor. Additionally, the used peripherals, such as timers, GPIOs or communication interfaces, will have an influence on the power consumption of the system;
2. The values in the data sheet are for a single microcontroller. In a smart sensor the SPU can be slightly different, because the manufacturer of the sensor adapted it to its need.

This wide range of parameters which influence the power consumption, make it necessary to calibrate the power model to achieve a reliable result.

Considering the timing of the measurements and the data calculated by the power model, Figure 10 shows a very good agreement. This makes the approach also usable for finding and debugging timing related behavior and error.

To achieve representative results, the power model for the SPU was calibrated with the values from the first experiments. The calculated mean values from the measurements, shown as green lines in the plot, can be found in Table 2. This table also shows the measured values for all other components.

**Figure 11:**
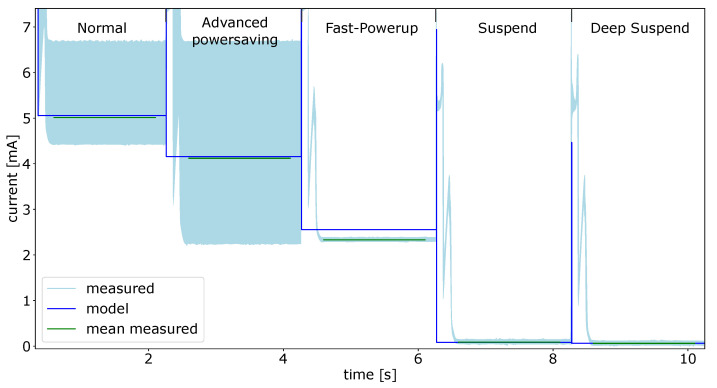
Gyro power modes model data and measured current values.

The results for the power modes of the gyroscope can be seen in Figure 11. As for all other component tests, each power state was enabled for two seconds before the SPU wakes up and reconfigures the gyro. The SPU was in standby mode during the measurements for the power consumption of the gyroscope. The measured values for each mode can be found in Table 2. To generate the graph shown in Figure 11, the standby current of the processing unit is removed from the offset data.

The power values from the data sheet fits the measured values, in most power states. Only the *fast power up* mode deviates from the data sheet values. For the gyroscope, it would be possible to work with the power estimates taken from the data sheet and gain reasonable results from the power model. However, for the test of the whole smart sensor, we took the measured data to adjust the power model. This ensures to achieve power estimates from the model, that are as close as possible to the real power dissipation of the system.

**Figure 12:**
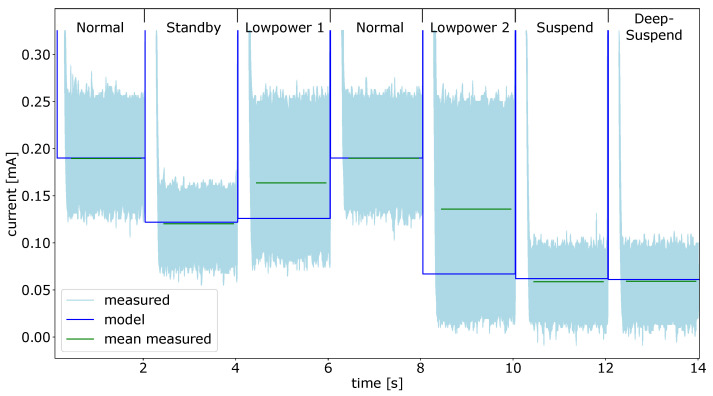
Accelerometer power modes model data and measured current values.

Figure 12 shows the measured current for all possible power states of the accelerometer. The measured values are as expected from the values of the data sheet for the most states. The third (lowpower 2) and the fifth (lowpower 1) mode differ from the expected values. This is because of the larger parameter set for these states. The developer can configure these modes with a variety of parameters to fit the desired purpose. The values in the data sheet are the lowest possible power consumption for these modes. For the experiments, we used the standard configuration for each mode. This leads to a slightly higher power consumption.

**Figure 13:**
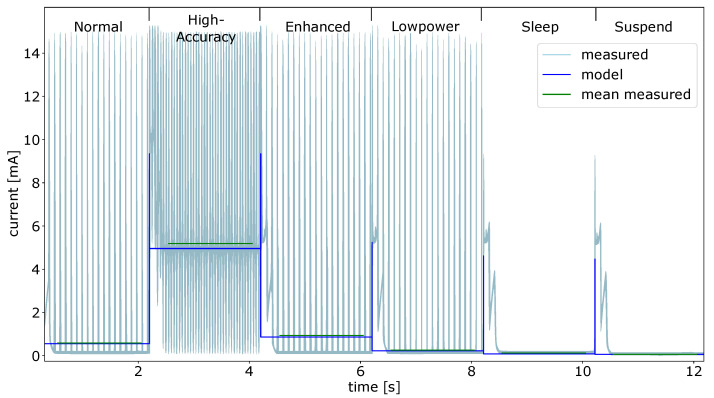
Magnetometer power modes model data and measured current values.

The current measurements for the magnetometer power modes are depicted in Figure 13. The mean values for all measured states are listed in Table 2. As for all other experiments, each mode was configured for 2 s. The mean of the measured current fits the data delivered by the power model. The high peaks of the measured signal are caused by the actual sampling of the magnetometer. So that each peak represents one sample, this behavior is not described by the PM. In the high accuracy mode, the measurements and the model data differs the most. As for all previews sensors, the data for the modeled power consumption are taken from the data sheet. These values are measured in perfect conditions and always the least power consumption possible.

The power values for each measured component of the smart sensor are listed in Table 2. This table contains the values for each individual power state of the particular component. We used these data to calibrate the power model before executing the final experiment.

### 6.2. Results for the Whole System

**Figure 14:**
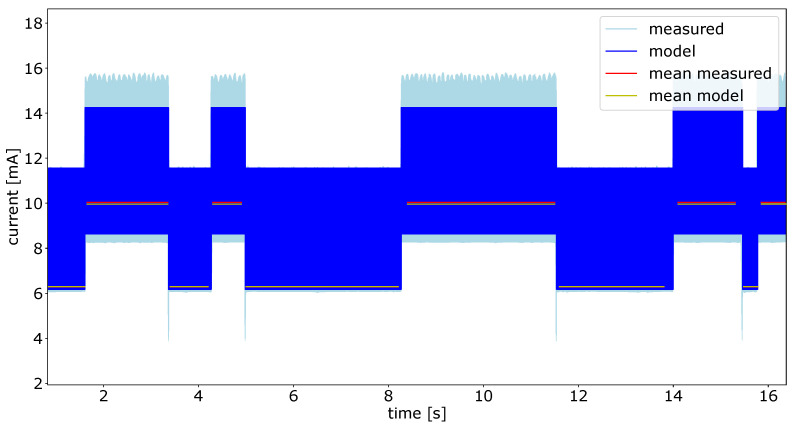
Power values during example test run.

After calibrating the power models of the Individual components, the complex test case experiment was conducted, as described in Section 5.2. The current values during this experiment are depicted in Figure 14. The actual measured values are shown in light blue, and the data delivered by the power model are in blue. To have a better comparison factor for both data, we calculated the mean for each segment. The mean values over the segments are shown in red for the measured data, and in yellow for the model data. As one can see in Figure 14, there are well-defined states over the runtime of the experiment. In this experiment, there are ten states of different length. The states with the overall lower power consumption are the *no-motion* states, where the sensor platform is not moved. In this state, only the accelerometer is running with a sample rate of 50 Hz. The SPU is waking up with a frequency of 50 Hz and performing the *any-motion* algorithm based on the accelerometer data. In the phases with the higher power consumption, the SPU is performing an orientation estimation based on the data of the gyroscope and the accelerometer. This calculation is executed with a frequency of 200 Hz

**Figure 15:**
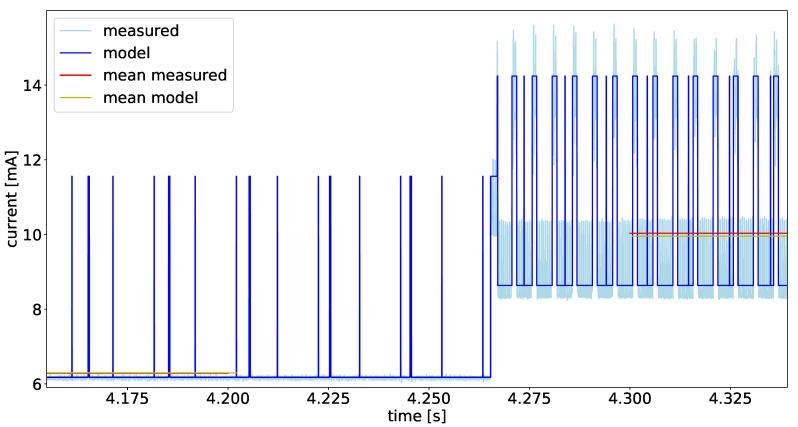
Zoomed view of the example test run in Figure 14.

Figure 15 shows a zoomed in view of the experiment for the whole system shown in Figure 14. A switch between two states can be seen in detail in this figure. It also shows the temporal accuracy between the measured data and the estimated power given by the model. This accurate timing enables the developer of the smart sensor firmware to easily find misbehavior in the program flow. Looking at Figure 15, one can identify several parts of the running firmware. In the first half of the signal, the wider peaks of the model signal are the 50 Hz wake-up, in these periods, the accelerometer is sampled and the *Any-Motion* algorithm is performed. The short peaks in the current are system timer interrupts triggered with a frequency of 100 Hz. The second part of the figure shows the system in the *motion* state. Here, the wide peaks are the sampling of all sensors, and the calculation of the orientation estimation algorithm followed by the *no-motion* algorithm. In this phase, the high peaks occur with a frequency of 200 Hz. The system timer interrupt does not change its frequency during the whole runtime of the firmware.

The mean current values for all ten segments can be found in Table 3. The table shows the mean values for the measured signal compared to the model data. The error is relative to the measured data in percentage for each individual signal.

It can be seen that the current data delivered from the power model fits the measured data in all phases. This applies for both the timing and the mean of the current amplitude. However, the model data differs in short time periods where the real measured current shows high peaks. This behavior in transitions is not described by the model. However, this is not necessary to achieve a good estimation of the mean power in usable time resolution.

In the *no-motion* sections with the lower sampling rate and lower activity, the model based current is slightly higher than the measured one. However, in the *motion* segments with higher sampling rate and more SPU activity the measured current is higher than the modeled one, which leads to a negative error. Considering all sections in the real-world-scenario experiment, the overall deviation between the measured data and the modeled data are less than 1%.

## 7. Conclusions

In the work at hand, we proposed an approach to enable the developer of smart sensor systems to observe the power consumption during the development process. These power consumption estimates can be observed in a live manner. Due to the possibility to see the power consumption live, it is easy to set these power consumption in relationship with specific parts of the firmware. Furthermore, the power estimates can be set in relationship to specific sensor data. In combination with the sensor-in-the-loop [3] approach, it is possible to investigate power consumption of smart sensor systems in a repeatable a reproducible way.

To show the usability of our approach, we created an example implementation on a BMF055 smart sensor. We developed a state based energy model which delivers the power estimates at runtime continuously. The power estimates of our model are derived from the data sheet of the corresponding sensor. To improve the accuracy of the power model, it is necessary to calibrate the model with previously measured power consumption.

The results show, that our approach is able to deliver live power estimates during the development process of smart sensor firmware. Furthermore, these estimates can be shown to be repeatable and reproducible during several runs of the firmware. This enables a developer of such firmware to debug the sensor system in an energy aware manner. The implemented approach, shows an average accuracy of 99% in estimating the power consumption for the complex real word scenario.

## Figures and Tables

**Table 1:** Power values from data sheets.

ATSAMD20J18 all in μA @3.3 V					
While1	NORMAL	IDLE0	IDLE1	IDLE2	Standby
2330	4030	1350	950	780	4
BMA280 all in μA @2.4 V					
Normal	Suspend	Deep Suspend	LowPower1	LowPower2	Standby
130	2.1	1	6.5	66	62
BMG160 all μA @2.4 V					
Normal	FastPowerUp	Suspend	DeepSuspend		
5000	2500	25	5		
BMM150 all in μA @ 2.4 V					
Normal	Normal @10 Hz	LowPower @10 Hz	High acc @20 Hz	Suspend	
800	500	170	4900	3	

**Table 2:** Measured power values.

ATSAMD20J18 all in μA @3.3 V					
NORMAL	IDLE0	IDLE1	IDLE2	Standby	
5170	3554	3081	1921	64	
BMA280 all in μA @3.3 V					
Normal	Suspend	Deep Suspend	LowPower1	LowPower2	Standby
190	59	59	136	164	120
BMG160 all μA @3.3 V					
Normal	AdvPowSave	FastPowerUp	Suspend	DeepSuspend	
5014	4120	2330	87	60	
BMM150 all in μA @ 3.3 V					
Enhanced @10 Hz	Normal @10 Hz	LowPower @ 10 Hz	High acc @20 Hz	Sleep	Suspend
936	588	254	5187	122	59

**Table 3:** Real-world example measurements and error.

Segment	Measured [mA]	Model [mA]	Error [%]
1	6.282	6.293	0.17
2	10.037	9.959	−0.77
3	6.284	6.293	0.15
4	10.035	9.958	−0.77
5	6.284	6.294	0.16
6	10.039	9.963	−0.75
7	6.283	6.294	0.19
8	10.038	9.959	−0.78
9	6.286	6.295	0.14
10	10.035	9.956	−0.79

## Abbreviations

The following abbreviations are used in this manuscript:

μC	Microcontroller
ADC	Analog Digital Converter
CI	Communication Interface
GPIO	General Purpose Input Output
IDE	Integrated Development Environment
PM	power model
SiL	Sensor-in-the-Loop
SPU	Sensor Processing Unit

## References

[1] Raghunathan V., Chou P.H. (2006). Design and Power Management of Energy Harvesting Embedded Systems. Proceedings of the 2006 International Symposium on Low Power Electronics and Design.

[2] Guo C., Ci S., Zhou Y., Yang Y. (2021). A Survey of Energy Consumption Measurement in Embedded Systems. IEEE Access.

[3] Gis D., Buscher N., Haubelt C. Advanced Debugging Architecture for Smart Inertial Sensors using Sensor-in-the-Loop. Proceedings of the 2020 International Workshop on Rapid System Prototyping (RSP).

[4] Microchip (2019). Power Debugger. https://www.microchip.com/DevelopmentTools/ProductDetails/ATPOWERDEBUGGER.

[5] Chen L.B., Chen Y.L., Huang I.J. (2011). A Real-Time Power Analysis Platform for Power-Aware Embedded System Development. J. Inf. Sci. Eng..

[6] Rudolf J., Gis D., Stieber S., Haubelt C., Dorsch R. SystemC Power Profiling for IoT Device Firmware using Runtime Configurable Models. Proceedings of the 2019 8th Mediterranean Conference on Embedded Computing (MECO).

[7] Simunic T., Benini L., De Micheli G. Cycle-accurate simulation of energy consumption in embedded systems. Proceedings of the 1999 Design Automation Conference (Cat. No. 99CH36361).

[8] Grüttner K., Hartmann P.A., Fandrey T., Hylla K., Lorenz D., Stattelmann S., Sander B., Bringmann O., Nebel W., Rosenstiel W. An ESL timing amp; power estimation and simulation framework for heterogeneous socs. Proceedings of the 2014 International Conference on Embedded Computer Systems: Architectures, Modeling, and Simulation (SAMOS XIV).

[9] Tiwari V., Lee M.T.C. (1995). Power Analysis of a 32-Bit Embedded Microcontroller. Proceedings of the 1995 Asia and South Pacific Design Automation Conference.

[10] Dochia R., Bogdan D., Burileanu C. Model for software power estimation of an 8-bit microcontroller. Proceedings of the CAS 2011 International Semiconductor Conference.

[11] Pallister J., Kerrison S., Morse J., Eder K. Data Dependent Energy Modeling for Worst Case Energy Consumption Analysis. Proceedings of the 20th International Workshop on Software and Compilers for Embedded Systems.

[12] NXP Semiconductors KINETIS-PET: Kinetis Power Estimation Tool. https://www.nxp.com/products/processors-and-microcontrollers/arm-microcontrollers/general-purpose-mcus/kv-series-cortex-m4-m0-plus-m7/kinetis-power-estimation-tool:KINETIS-PET.

[13] Microchip Technology Inc Power Debugging Module. https://onlinedocs.microchip.com/pr/GUID-F897CF19-8EAC-457A-BE11-86BDAC9B59CF-en-US-10/index.html?GUID-590491AB-3C05-4C73-B4D4-DD739B095630.

[14] Bosch Sensortec Data Sheet BMF055 Custom Programmable 9-Axis Motion Sensor. https://www.bosch-sensortec.com/media/boschsensortec/downloads/datasheets/bst-bmf055-ds000.pdf.

[15] Bosch Sensortec BMA280 Digital, Triaxial Acceleration Sensor. https://www.bosch-sensortec.com/media/boschsensortec/downloads/datasheets/bst-bma280-ds000.pdf.

[16] Bosch Sensortec BMG160 Digital, Triaxial Gyroscope Sensor. https://media.digikey.com/pdf/Data%20Sheets/Bosch/BMG160.pdf.

[17] Bosch Sensortec BMM150 Geomagnetic Sensor. https://www.bosch-sensortec.com/media/boschsensortec/downloads/datasheets/bst-bmm150-ds001.pdf.

[18] Bosch Sensortec Bosch Sensortec Sensor Drivers. https://www.bosch-sensortec.com/software-tools/software/drivers/.

[19] Datatec (2019). CX3300A Series Device Current Waveform Analyzer. https://www.datatec.de/media/pdf/4c/74/02/Keysight_CX3300A.pdf.

[20] Microchip Technology ATSAMD20J18—Arm Cortex-M3 MCU. https://www.microchip.com/wwwproducts/en/ATSAMD20J18.

[21] Madgwick S.O.H., Harrison A.J.L., Vaidyanathan R. Estimation of IMU and MARG orientation using a gradient descent algorithm. Proceedings of the 2011 IEEE International Conference on Rehabilitation Robotics.

[22] (2021). STMicroelectronics Optimizing Power and Performance with STM32L4 and STM32L4+ Series Microcontrollers AN4746. https://www.st.com/content/ccc/resource/technical/document/application_note/5c/cb/90/97/4b/84/4e/81/DM00216518.pdf/files/DM00216518.pdf/jcr:content/translations/en.DM00216518.pdf.

[23] NXP Semiconductors (2016). MKW40Z Power Consumption Analysis. https://www.nxp.com/docs/en/application-note/AN5272.pdf.

